# *Annamanum flavimaculatum*, a new species of longhorn beetle (Coleoptera, Cerambycidae) from China

**DOI:** 10.3897/zookeys.889.38296

**Published:** 2019-11-14

**Authors:** Shulin Yang, Shaoyong Yang

**Affiliations:** 1 School of Life Sciences, Guizhou Normal University, Guiyang, Guizhou 550001, China Guizhou Normal University Guiyang China; 2 Administrative Office of the Leigongshan National Nature Reserve, Leishan, Guizhou 557100, China Leigongshan National Nature Reserve Leishan China

**Keywords:** Lamiinae, Leigongshan Nature Reserve, Lingui, Maoershan

## Abstract

*Annamanum flavimaculatum***sp. nov.** is described and illustrated from Guizhou and Guangxi, China. Diagnosis for distinguishing the new species to its close congeners is presented and identification key to the genus is also updated.

## Introduction

The genus *Annamanum* Pic, 1925 is a large genus in the subfamily Lamiinae (Coleoptera, Cerambycidae) with 30 described species (Tavakilian and Chevillotte 2018) distributed in South China, Japan, Vietnam, Laos, Cambodia, India, Myanmar, and Malaysia (Lin and Ge 2017). Of these, 14 species were recorded in China (Lin and Ge 2017; Holzschuh 2017; Tavakilian and Chevillotte 2018). With the specimens collected from Leigonshan area of Leishan County, Guizhou Province, and Maoershan and Lingui, Guangxi Province, China, a new species of the genus is discovered and described as *Annamanum flavimaculatum* in this article. In addition to *A. albisparsum* (Gahan, 1888), *A. lunulatum* (Pic, 1934), and *A. magnum* Holzschuh, 2017, this is the fourth *Annamanum* species recorded in the Leigongshan area.

## Materials and methods

Specimens were collected by two collecting methods: net sweeping and six level Lindgren funnel traps (Sanyong Biologic Techonology Ltd, Xiamen, Fujian Province, China) with 99% ethanol as lure. Collected specimens were pinned or glued on pinned paper cards. Labels were handwritten or printed in Chinese. Materials from Guizhou are preserved in the School of Life Sciences, Guizhou Normal University, Guiyang, Guizhou, China. Materials from Guangxi are preserved in Collection of Wen-Xuan Bi, Shanghai, China.

Specimen examination and dissection were conducted under an AmScope SM-4TZ stereomicroscope. Adults were photographed with Canon EOS 6D digital camera equipped with EOS MP-E 65 lenses. Male genitalia were photographed with Olympus DP22 camera mounted on an Olympus SZX7 stereomicroscope.

The collection acronyms used in the text are as follows:

**CBWX** Collection Wen-Xuan Bi, Shanghai, China;

**GZNULS**School of Life Sciences, Guizhou Normal University, Guiyang, China.

## Taxonomy

### Annamanum flavimaculatum sp. nov.

Taxon classificationAnimaliaColeopteraCerambycidae

BF6CC0CF-7653-53CB-975F-C1349AD10D3C

http://zoobank.org/C4EE8EDD-377D-4625-94DB-24C6FA355C94

Figures 1 , 2 , 3

#### Type locality.

Queniao Tea Farm, Queniao Village, Leishan County, Guizhou Province, China.

#### Type-specimen.

Holotype male, glued on paper point, with genitalia in a separate microvial. Original label: “中国贵州省雷山县方祥乡雀鸟村茶场，2015年6月18日，六层漏斗诱捕器，杨书林采” [Queniao Tea Farm, Queniao Village, Fangxiang, Leishan County, Guizhou Province, China, 2015.VI.18, six level Lindgren funnel trap, Shulin Yang leg. (GZNULS)], HOLOTYPE / Annamanum / flavimaculatum / Shulin Yang [handwritten red label].

#### Other materials.

Paratypes: 2♂♂, original labels: “中国贵州省雷山县雷公山国家级自然保护区，2012年7月22日，2016年7月13日，杨书林采” [Leigongshan National Nature Reserve, Leishan County, Guizhou Province, China, collecting dates: 2012.VII.22 and 2016.VII.13, Shulin Yang leg.] (GZNULS); 2♂♂, original labels: “中国贵州省雷山县雷公山国家自然保护区，2017年7月22~27日，李泊言采” [Leigongshan National Nature Reserve, Leishan County, Guizhou Province, China, 2017.VII.22~27, Boyan Li leg.] (GZNULS); 1♀, original labels: “中国贵州省雷山县雷公山国家自然保护区，2017年8月27日，杨绍勇采” [Leigongshan National Nature Reserve, Leishan County, Guizhou Province, China, 2016.VIII.27, leg. Shaoyong Yang] (GZNULS); 1♀, Original label: “中国贵州省雷山县方祥乡雀鸟村茶场，2015年6月18日，杨光祖采” [Queniao Tea Farm, Queniao Village, Leishan County, Guizhou Province, China, 2015.VI.18, Guangzu Yang leg.] (GZNULS); 1♀, Original label: “中国贵州省雷山县方祥乡雀鸟村茶场，2019年8月2日，杨书林采” [Queniao Tea Farm, Queniao Village, Leishan County, Guizhou Province, China, 2019.VIII.2, Shulin Yang leg.] (GZNULS); 1♀, Original label: “中国贵州省雷山县方祥乡雀鸟村茶场，2019年9月12日，杨书林采” [Queniao Tea Farm, Queniao Village, Leishan County, Guizhou Province, China, 2019.IX.12, Shulin Yang leg.] (GZNULS); 1♂, original labels: “广西猫儿山迴龙寺，1900 – 1700 m, 2012.VII.23，毕文烜” [Translation: Huilongsi, Maoershan, Guangxi Province, 2012.VII.23, Wen-Xuan Bi leg.] (CBWX); 1♂, original labels: “广西猫儿山三江源，1950–2000 m, 2014.VII.30，宋晓彬” [Sanjiangyuan, Maoershan, Guangxi Province, 2014.VII.30, Xiaobin Song leg.] (CBWX); 1♀, original labels: “广西临桂广福顶，1350 m, 2018.VII.2” [Guangfuding, Lingui, Guangxi Province, 2018.VII.2, local collector leg.] (CBWX).

**Figure 1.**
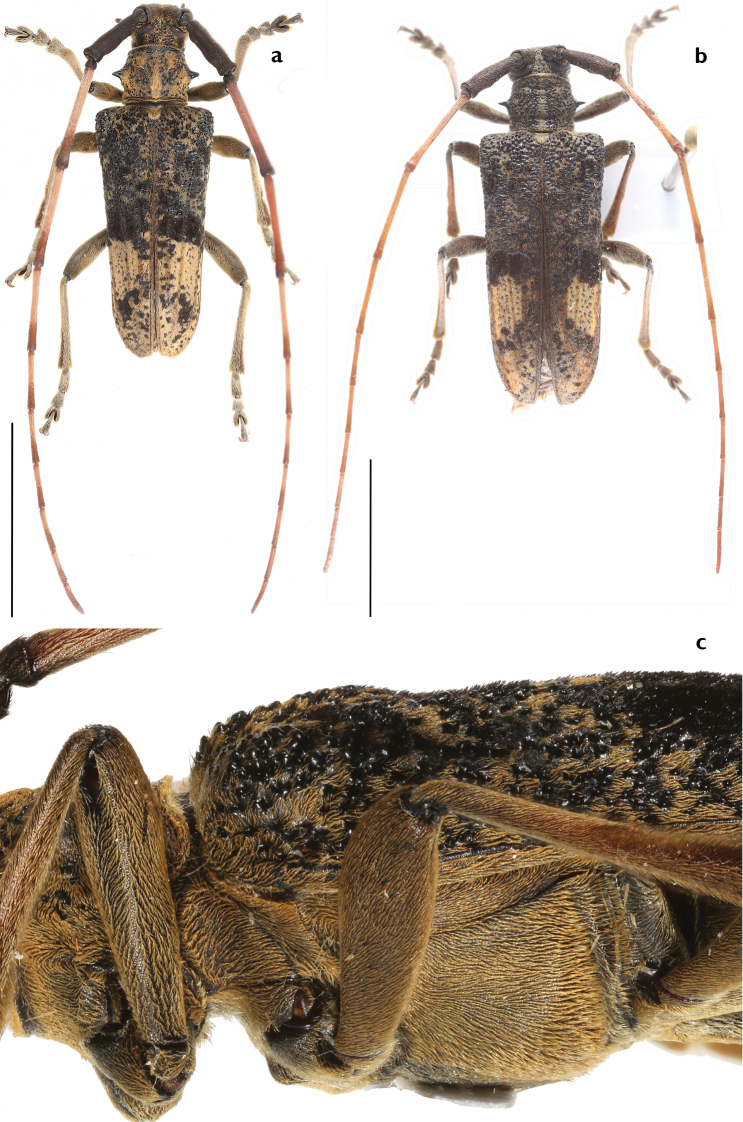
Habitus of *A. flavimaculatum* sp. nov. **a, c** male **b** female (**a, b** dorsal view **c** lateral view showing the mesosternal intercoxal process; not to scale). Scale bars: 10 mm (**a, b)**.

#### Differential diagnosis.

The new species can be distinguished from its congeners by its unique elytral pattern: apical half of elytron mostly covered with dense yellow hairs that compose a large yellow marking; black hair clustered as dots near suture on the anterior half of the yellow marking, sometimes weakly forming a line, and then obliquely extending from suture backwards to elytral margin; small black hair clusters sparsely scattered within the yellow marking.

**Figure 2.**
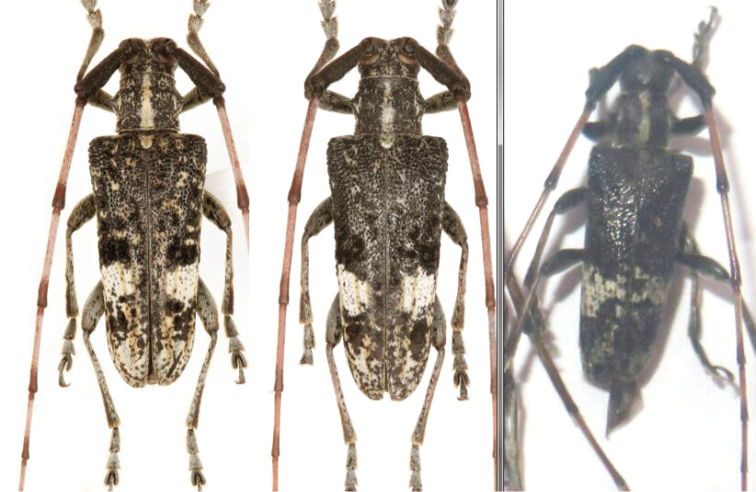
Habitus of *A. flavimaculatum* sp. nov. of specimens collected from Guangxi, China (Photographs courtesy of Mei-Ying Lin and Wen-Xuan Bi).

#### Description.

**Figure 3.**
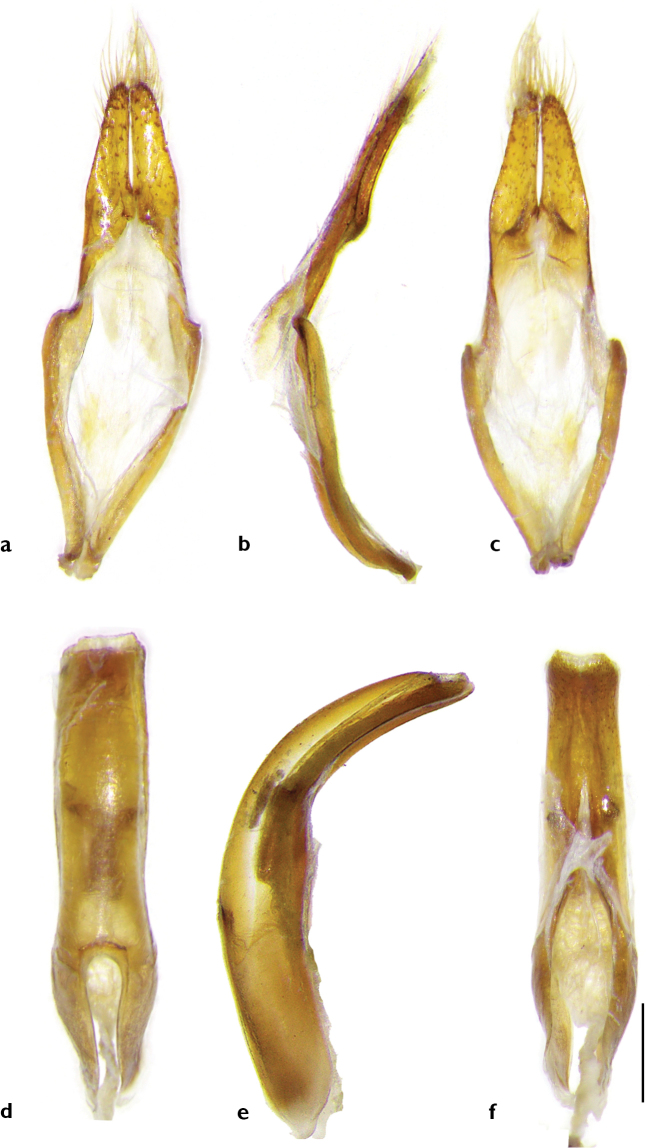
Male genitalia of *A. flavimaculatum* sp. nov. **a–c** tegmen **d–f** median lobe (**a, c** dorsal view **b, e** lateral view **c, f** ventral view). Scale bar: 0.5 mm.

**Figure 4.**
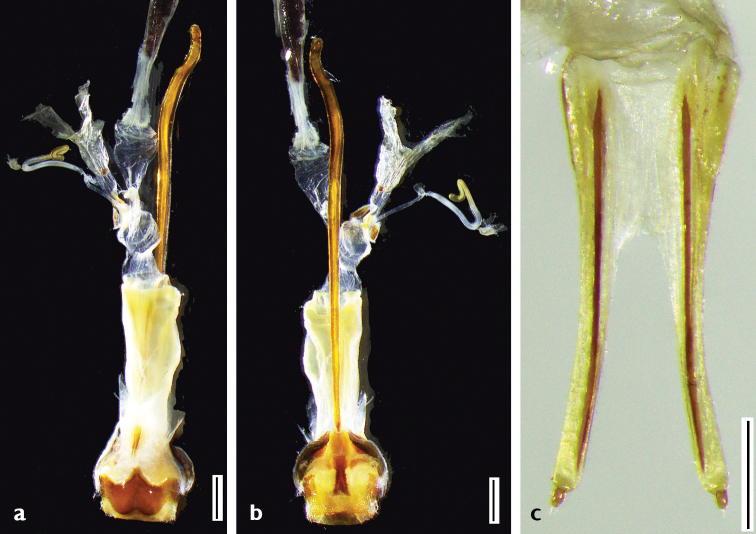
Female terminalia and genitalia of *A. flavimaculatum* sp. nov. **a** dorsal view **b** ventral view **c** ovipositor (dorsal view). Scale bars: 1 mm (**a, b**) 0.5 mm (**c**).

***Body length***: 13.8–20.4 mm, male (Figure 1a, c) 13.8–17.6 mm (*N* = 5), female (Figure 1b) 17.5–20.4 mm (*N* = 4). Measurements for specimens from Guanxi (Figure 2) not available. ***Head***: black, frons generally densely punctured, vertex densely punctured, both frons and vertex covered with dense yellow hairs. Antennae of males exceed apex of elytra by six antennomeres; of females by five antennomeres; antennal tubercles strongly raised; scape and pedicel black, covered with long hairs, not erect but flat towards apex; cicatrix complete, narrow; rest of the antennomeres reddish brown, sparsely covered with white yellowish hairs; base and apex of each antennomere are covered with darker hairs. Eyes deeply emarginated; lower lobe twice as high as gena and one-fourth as wide as frons width between lower lobes of eyes. Labium with small sparse punctures and sparse long dark brown hairs. Mandibles with dense long yellow hairs at outer side and sparse hairs on the front. ***Thorax***: Pronotum black with coarse granules, covered with yellow hairs, whose thickness varies among individuals; disk slightly raised; a small callus at each side of the apical margin, not extending beyond middle. Lateral spines strong, acute, slightly posteriorly and upwards curved. Sternum reddish brown, covered with dense yellow hairs. Scutellum covered with dense white yellowish pubescence, apex rounded. Mesosternal intercoxal process with a slightly projected antero-ventral tubercle (Figure 1c). ***Elytra***: gradually tapered in male, less tapered in female, with irregular coarse granules obliquely protruding backwards and gradually smaller posteriorly. Basal fifth bulged between humeri and scutellum. Basal half black, intermingled with yellow and black hairs; black hairs forming a broad transverse black band at the middle, nearly reaching suture. Apical half reddish brown, without granules but with coarse small punctures, mostly covered by yellow hairs forming a large yellow marking with intermingled black hair dots; some of these dots are near suture on the anterior half of the yellow marking and weakly form a line which extends obliquely towards outer margin of elytron from middle of the marking; black dots larger in female. Apex nearly rounded, slightly truncated in inner half. ***Legs***: with dense white yellowish pubescence; femora dark brown, clubbed, not cylindrical, with sparse small punctures; tibiae reddish brown. ***Abdomen***: reddish brown, ventrites with white yellowish pubescence intermingled with sparse punctures. Pygidium shallowly truncated at apex, deeper at the middle in some males. The sexual dimorphism is not very conspicuous. ***Male genitalia*** (Figure 3): Tegmen (Figure 3a–c), lateral lobes gradually narrowing towards apices, each apex rounded with setae that are shorter than half of lateral lobe. Median lobe (Figure 3d–f) moderately curved, median struts about half the length of median lobe, ventral plate truncated at apex, slightly concave in the middle. ***Female genitalia*** (Figure 4): bursa copulatrix and spermatheca small and short; spermathecal duct and spermathecal gland long, apex of spermathecal gland winded up like a spiral, but with the winding up part not completing a circle.

#### Etymology.

The name refers to the yellow patch on the apical half of elytron.

#### Distribution.

China: Guizhou, Guangxi.

### Modified couplets to key by Gressitt (1951) of Chinese *Annamanum* species to accommodate the new species

**Table:** 

1	Pronotum without three distinctly raised areas and without a prominent tooth on each side anterior to lateral tubercle	**2**
–	Above characters present; elytra dark brown with several oblique or irregular pale brown stripes or marks	***A. sinicum***
2	Elytra each without a large blackish brown or black lateral spot behind middle	**3**
–	Elytra each with a large blackish brown or black lateral spot behind middle	**7**
3	Elytra without yellow bands crossing on suture or yellow patches	**5**
–	Elytra with yellow bands crossing on suture or yellow patches	**4**
4	Elytra with two yellow bands crossing on suture, forming a shape of letter x	***A. yunnanum***
–	Elytra with one big yellow marking at apex, not forming a shape of letter "X"	***A. flavimaculatum***
5	Elytra each with a fairly large yellow or whitish spot	**6**
–	Elytra with many small yellow or whitish marks, sometimes forming a vague band at middle, but no very large spots	***A. albisparsum***
6	Basal antennal segments with long slender hairs; frons coarsely granulate	***A. strandi***
–	Basal antennal segments without long slender hairs; frons not coarsely granulate; antennae uniformly pubescent	***A. szetschuanicum***
7	The lateral spot on elytra black, largest width about 1/4 of elytral length, bounded by a thin ring of yellow hairs, these yellow hairs not expanding anteriorly and posteriorly	***A. lunulatum***
–	The lateral spot on elytra velvety brown, largest width about 1/3 of elytral length, with pale and yellow hairs around, these hairs expanding anteriorly to basal 1/4 of elytra and posteriorly to apices of elytra	***A. magnum***

## Supplementary Material

XML Treatment for Annamanum flavimaculatum
